# Pioneering Experience of Uniportal Video-Assisted Thoracoscopic Surgery for Anterior Release of Severe Thoracic Scoliosis

**DOI:** 10.1038/s41598-020-57984-x

**Published:** 2020-01-21

**Authors:** Cheng-Min Hsu, Kuan-Wen Wu, Mong-Wei Lin, Ken N. Kuo, Jia-Feng Chang, Ting-Ming Wang

**Affiliations:** 1 Department of Orthopaedic Surgery, National Taiwan University Hospital, Taipei, 100 Taiwan; 2 Department of Orthopaedic Surgery, Linkou Chang Gung Memorial Hospital, Chang Gung University, Taoyuan, 333 Taiwan; 3 Department of Thoracic Surgery, National Taiwan University Hospital, Taipei, 100 Taiwan; 4 Cochrane Taiwan, Taipei Medical University Hospital, Taipei, 110 Taiwan; 5 Department of Internal Medicine, Shuang Ho Hospital, New Taipei, 235 Taiwan; 6 Graduate Institute of Aerospace and Undersea Medicine, Department of Medicine, National Defence Medical Center, Taipei, 114 Taiwan

**Keywords:** Orthopaedics, Outcomes research, Musculoskeletal system

## Abstract

The optimal way to treat severe thoracic scoliosis remains controversial. Compared with conventional procedures, the uniportal video-assisted thoracoscopic surgery (UniVATS) rises in popularity in thoracic surgery because of less pain and faster recovery. This retrospective study aimed to apply UniVATS to treat severe thoracic scoliosis. Between October 2013 and March 2018, eight scoliotic patients with extremely large Cobb angle and profoundly limited flexibility underwent UniVATS for anterior release, followed by posterior instrumentation and fusion. The mean age at the time of surgery was 14.8 ± 2.4 years and the mean follow-up was 2.2 ± 1.3 years. The average levels of anterior thoracic discectomy and posterior fusion were 3.6 ± 0.7 and 11.5 ± 1.2, respectively. The mean coronal and sagittal correction rates were 70 ± 19% and 71 ± 23%, respectively. UniVATS contributed to minor access trauma (3-cm incision) with minimal blood loss, shorter operation time (75 ± 13 mins), less requirement of stay in the intensive care unit (0.3 ± 0.5 day) or chest tube placement (0.3 ± 0.7 day), speedier and narcotic-free recovery, and earlier ambulation within one day. This is the first study to assess the safety and efficacy of UniVATS in the treatment of severely stiff thoracic scoliosis, providing comparable surgical outcomes, less pain, faster recovery and superior cosmetic results without significant complications.

## Introduction

Scoliosis is a three-dimensional deformation of the spine. The goal of operative treatment is to prevent long-term scoliosis progression by effective correction of the coronal, sagittal, and rotational deformities with minimal fusion levels, maintaining appropriate coronal and sagittal balance^1^. Posterior spinal fusion with instrumentation (PSF/PI) is the mainstay of surgical treatment for scoliosis^1^, and it is usually offered by the surgeons when the Cobb angle ≥45°. Several studies^2–4^ demonstrated the superiority of pedicle screws fixation over other posterior instrument systems, such as hybrid hooks and screws or all-hooks instrumentation, providing stronger three-column purchase of vertebral segments with better correction and stabilization.

For those patients with a severe and stiff curve ≥70° and the flexibility ≤30% (or residual curve on bending films ≥50°)^5,6^, the best choice of surgical approach is still controversial. The PSF/PI in severe cases is fraught with risks and limitations of correction. Therefore, a search for better management is required. Some reinforcements were considered, for example, (1) an anterior release beforehand^6^, (2) staged posterior operation, (3) osteotomy, such as Smith-Petersen osteotomy (SPO), pedicle subtraction osteotomy (PSO), or vertebral column resection (VCR), (4) traction methods, such as preoperative halo-gravity traction, intraoperative halo-femoral traction^7^. Among the above methods, the staged anterior release and PSF/PI capable of three-dimensional correction with increased flexibility were most adopted. In the past, the anterior release was performed through an open thoracotomy. Nonetheless, it was later replaced by multi-portal video-assisted thoracoscopic surgery (VATS) because of relative minor invasiveness with equal therapeutic effectiveness. The safety and efficacy of multi-portal VATS for anterior approach were later proven by Lenke^6^ and Bullmann *et al*.^8^, recommended the relative indications for severe, stiff curves, severe lordosis or hypokyphosis, and severely immature patients^9^. In a biomechanical cadaveric study, Wollowick^10^ showed that anterior release generated significantly more thoracic rotation than posterior osteotomy and maximized three-dimensional correction. However, the invention of pedicle screw largely increases the structural stability of PSF/PI, and there is a doubt on the necessity of adding an anterior approach for severe cases. Several studies^11–13^ have demonstrated that two-stage anterior/posterior surgery has only a limited advantage in terms of correction, while the disadvantages and complications of anterior release could be avoided by the posterior-only approach. To sum up, the controversies exist whether the benefits outweigh the possible complications from the anterior operation.

To confirm the advantages of anterior approaches in severe cases, surgeons should update techniques. With the rapid development of thoracoscopy, the single incision of UniVATS shows a greater field of vision and higher resolution than the conventional multi-portal VATS. Recently, the popularity of this technique is increasing in the field of thoracic surgery for complicated procedures, including major pulmonary resections, carinal resections, or tracheobronchial reconstructions^14^. Previous studies^15,16^ have shown the UniVATS is non-inferior to conventional multi-portal VATS in the safety and therapeutic effects of lung cancer. Moreover, the UniVATS has potential advantages^15–18^ of reduced access trauma, less pain, fewer complication and faster recovery. Based on the benefits mentioned above, we have implemented the technique since 2013. Our study aimed to compare UniVATS with conventional procedures in the safety and efficacy of surgical treatment for patients with severe and stiff scoliosis.

## Material and Method

The study had been approved by the Research Ethics Review Committee at National Taiwan University Hospital (201812024RINC) in accordance with the Good Clinical Practice guidelines and governmental laws and regulations. The informed consent was obtained from all participants or their legal guardians. Between October 2013 and March 2018, eight scoliosis patients (six girls and two boys) with Cobb angle more than 85° (range, 85–117°) and limited flexibility with bending radiographs more than 70° (range, 70–100°; recovery rate 11 ± 7%) were selected. The aetiology of the curves included seven cases of adolescent idiopathic scoliosis (AIS) and one case of Beals syndrome. The average age was 14.8 ± 2.4 years old.

A two-staged surgery was performed, including anterior release followed by a posterior instrumented correction in two days. At the first stage of surgery, UniVATS for anterior release was performed under general endotracheal anaesthesia with a double-lumen tube to achieve single-lung ventilation. The patient was placed in the lateral decubitus position with the thoracic convex side up, and an axillary roll was placed under the downside. The placement of the incision was crucial in light of curvature types, the distance between the apex and the chest wall, and lung abnormalities if any. After the C-arm localization, a small incision was usually made at the intercostal space on the middle axillary line toward the apex of the curvature. A 5-mm 30-degree thoracoscopy (Olympus) was inserted in the posterior part of the incision to obtain the most exquisite anatomic view. To maintain the position of the camera posteriorly to the patient, a suture around the camera to pull it back was suggested^16^. The surgeon and the assistants were all positioned in front of the patient to have the same and appropriate surgical view.

**Figure 1:**
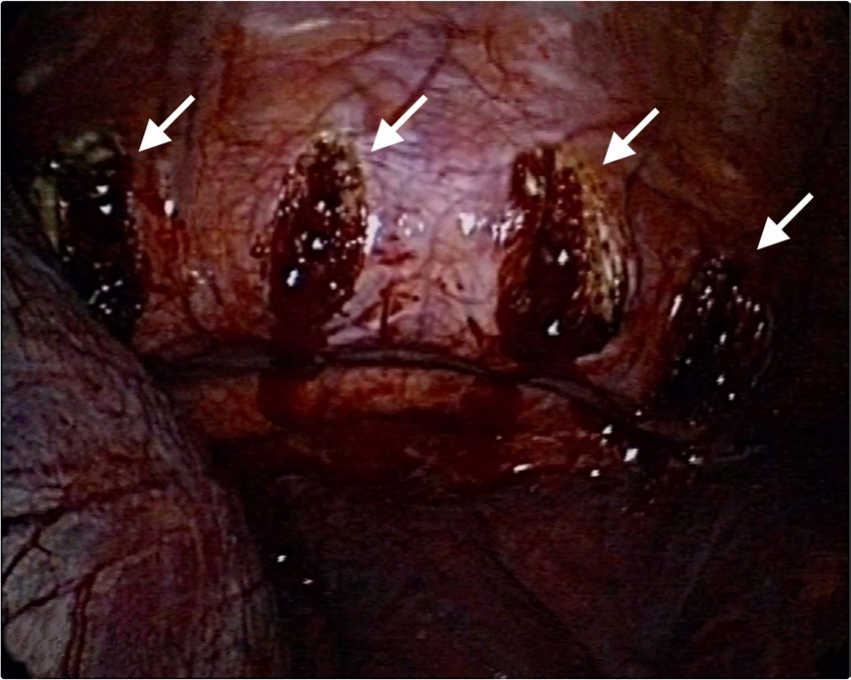
The perioperative thoracoscopic view for anterior release showed four levels of discectomy (arrow) and the release of corresponding anterior longitudinal ligaments.

After the lung deflated, the sympathetic trunk and artery of Adamkiewicz were identified and protected. The discs and end-plates of selected levels were removed, and their corresponding anterior longitudinal ligaments (ALL) were released (Fig. 1). After meticulous haemostasis, the intercostal nerve blockade was performed for better postoperative pain control by injection of 1.5 ml 0.5% bupivacaine beneath the parietal pleura for each intercostal space. Prior to wound closure, a combination waste vent (CWV) drain was placed through the camera port to check for possible air leakage and then removed. Most patients did not receive chest tube placement at the time of surgery, because the drainless protocol of UniVATS suggested by thoracic surgeons contributed to better recovery. Patients were encouraged to ambulate on the following day, and postoperative narcotic was not given routinely.

The second-stage was performed two days later. There was no need for traction between two stages of surgery. Thoracic apical Smith-Petersen osteotomy was performed in all cases for better coronal correction. The posterior pedicle screw instrumentation was performed to correct the deformities through de-rotation and distraction-compression manoeuvring followed by fusion with autogenous bone graft with additional artificial graft if necessary.

The preoperative radiographs, including the coronal/sagittal view in standing position, both sides bending films and three-dimensional CT reconstruction of the whole spine were obtained for evaluation. The postoperative radiographs of coronal/sagittal view in the standing position were obtained at the latest follow up. While recording radiographic findings, the clinician was blinded to the functional and perioperative data. Deformities were measured on coronal and sagittal films using the Cobb method. Calculations for curve magnitude and percent correction were recorded using the worst deformity values. Thoracic kyphosis was measured from T3–T12. Normal thoracic kyphosis at the age between 10–19 was considered to be 26°^19^. In calculating percentage correction for hypokyphosis cases, the following formula was used: Percentage of hypokyphosis correction = [100 × (postoperative kyphosis − preoperative kyphosis)/(26 − preoperative kyphosis)].

## Results

**Figure 2:**
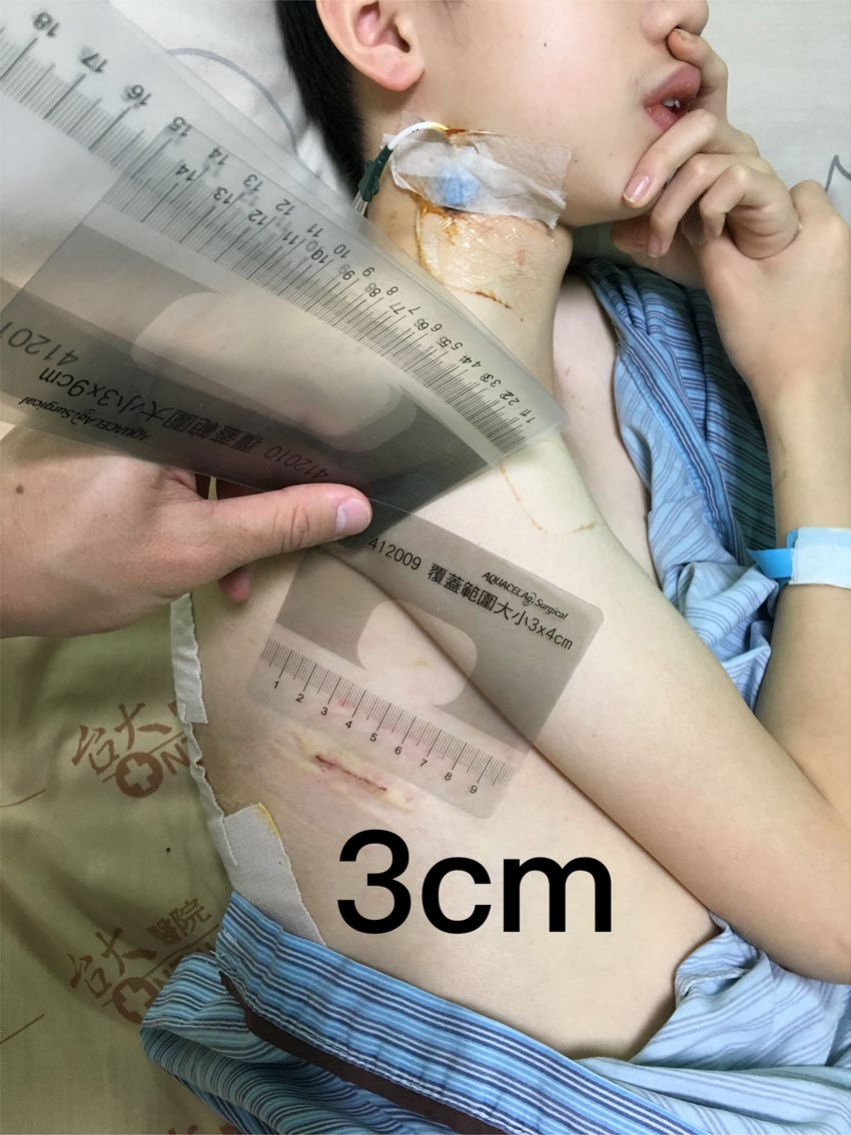
The 3-cm incision wound in anterior approach of UniVATS was documented after the second-stage posterior operation.

All of the eight patients who underwent index procedures were followed up at clinics for 2.2 ± 1.3 years. The average levels of anterior thoracic discectomy and posterior fusion were 3.6 ± 0.7 and 11.5 ± 1.2, respectively. The average first stage anterior operation time was 75 ± 13 minutes with minimal blood loss. The average incision wound of the UniVATS was 3 ± 0.5 cm (Fig. 2). Table 1 demonstrated the preoperative and postoperative comparison of patients with severe scoliosis underwent UniVATS in staged anterior-posterior approach. The mean preoperative major thoracic curve was 94 ± 11°, and the mean postoperative correction angle was 29 ± 18° with a correction rate of 70 ± 19%. In addition to coronal correction, sagittal thoracic hypokyphosis was corrected from 3.0 ± 1.4° to 20 ± 4.9° with a correction rate of 71 ± 23%.

**Table 1:** The preoperative and postoperative comparison of patients with severe scoliosis underwent the staged UniVATS (ICU = intensive care unit).

Patient data	Total number	8
	Age	14.8 ± 2.4
	Male: Female	2:6
Anterior disc removal		3.6 ± 0.7
Posterior fusion level		11.5 ± 1.2
Scoliosis	Preop (°)	94 ± 11
	Postop (°)	29 ± 18
	Correction rate (%)	70 ± 19
**Hypokyphosis**	Preop (°)	3 ± 1.4
	Postop (°)	20 ± 4.9
	Correction rate (%)	71 ± 23
Operation time (mins)		75 ± 13
Blood loss		Minimal
Total complication, **n (%)**		1 (13%)
Recovery (days)	ICU stay	0.25 ± 0.46
	Chest tube placement	0.25 ± 0.71

Because of less assess trauma and narcotic-free recovery, our patients ambulated in one day after UniVATS. Two of the eight patients required one-night stay in the intensive care unit (ICU) due to underlying Tetralogy of Fallot (TOF) and postoperative pneumohemothorax, respectively. The postoperative pneumohemothorax was noted in the first postoperative day and managed by an 8-french pigtail drain tube. No further surgical intervention was required. Both of them recovered well and were transferred to the general ward on the following day. There was no need of analgesics 2 weeks postoperatively, and no chronic pain noticed. One of the cases was presented as followed.

**Figure 3:**
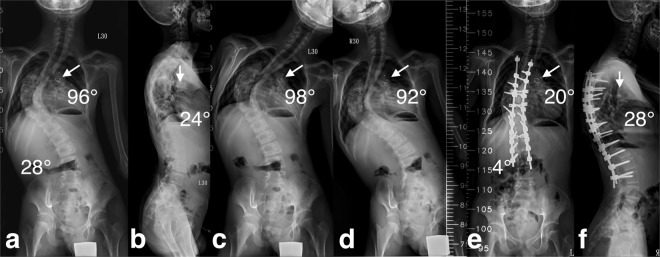
A 16-year-old boy had underlying diseases of TOF and severe AIS. The preoperative radiographs showed severe scoliosis with limited flexibility, and postoperative radiographs showed significant improvements in the deformities. The pulmonary stent and Cobb angles of each curve were designated. (**a**) Preoperative AP radiograph; (**b**) Preoperative lateral radiograph; (**c**,**d**) Preoperative left and right bending films; (**e**) AP radiograph at latest follow-up; (**f**) Lateral radiograph at latest follow-up.

This is a 16-year-old boy with underlying TOF, growth hormone deficiency and severe AIS. He received total correction surgery for TOF at the age of 11 months and pulmonary stent insertion (Arrows on Fig. 3) for pulmonary stenosis at the age of 10 years. The growth hormone deficiency was treated with Genotropin, and the measured skeletal bone age was 13.5 years old with Risser sign 1. The rapid progression of AIS led to severe back pain and restrictive ventilatory defect, which forced us to consider corrective surgery before skeletal maturity. His thoracic and lumbar spine deformities were 96° and 28° (Fig. 3a), respectively. The residual thoracic curve on the side bending film was 92° (Fig. 3c,d), only with 4% improvement, indicative of poor flexibility. After discussion with paediatricians, cardiovascular surgeons, thoracic surgeons and anaesthesiologists, the two-stage surgery was the treatment of choice, including UniVATS for anterior releases (T7–11) and PSF/PI (T4–L3). The prior cardiovascular surgery adhesions would not interfere with the UniVATS. He had one-night stay in the ICU due to high cardiopulmonary risk and was discharged on the fifth postoperative day. At the latest postoperative follow-up (18 months postoperatively), he did not experience any discomfort, and the corrected curvature maintained. His thoracic and lumbar curves were corrected to 20° and 4°, indicating 79% and 86% improvement, respectively (Fig. 3e).

## Discussion

For those scoliosis patients with severe and stiff thoracic curves, choosing an optimal surgical approach is still controversial. The consensus from thoracic surgery specialty^15–18^ indicated that benefits of UniVATS included less pain, fewer complications and faster recovery. To confirm the advantages of anterior approaches in difficult cases, we intended to apply UniVATS in the treatment of profound and stiff thoracic scoliosis. This is a preliminary cohort study to compare the safety and surgical outcomes between this novel UniVATS technique with conventional anterior release procedures.

The surgical outcomes of UniVATS in our cases were not inferior to conventional anterior release approaches in the literature (Table 2). Peter O. Newton *et al*.^20^ evaluated 18 cases of open thoracotomy anterior release followed by posterior spinal fusion (with or without instrumentation). The percentage of coronal Cobb angle correction was 60% (range, 47–82%) with 16% complication rate. Arlet *et al*.^21^ performed a meta-analysis of 10 studies with a total of 151 cases received conventional multi-portal VATS and posterior spine fusion. The percentage of coronal Cobb angle correction was 55–63% with 18% complication rate. The most frequent complications were pulmonary events that required prolonged ventilator support. Al-Sayyad *et al*.^22^ reported that 70 cases received conventional multi-portal VATS for anterior release, followed by posterior spinal fusion and instrumentation. The percentage of coronal Cobb angle correction was 68% (range, 41–91%) with 11% complication rate, with pulmonary complications in 3 out of 8 patients. Table 2 summarized these studies, whereas, the kyphotic correction was not presented due to the heterogeneity in different investigations. The average levels of anterior release through conventional approaches were much more than that in our UniVATS technique (3.6 ± 0.7). However, a learning curve was warranted. The first case in our series had only three levels released. Through mastering the technique in a short time, our team could increase the levels of releasing.

**Table 2:** Comparison between conventional anterior approaches^20–22^ and UniVATS.

		Newton^20^	V Arlet^21^	Al-Sayyad^22^	Our institute
Operation method		Open	VATS	VATS	UniVATS
Total number		18	151	70	8
Anterior disc removal		6.1 ± 2.9	4–7	7.78 ± 1.57	3.6 ± 0.7
Scoliosis	Preop (°)	73 ± 18	65	72 ± 17	94 ± 11
	Postop (°)		25–37	24 ± 15	29 ± 18
	Correction rate (%)	60	56–63	68 ± 18	70 ± 19
Operation time (mins)		128 ± 39	184	256 ± 51	75 ± 13
Blood loss (ml)		270 ± 154	246	285 ± 256	Minimal
Recovery (days)	ICU stay	1.2 ± 3.5	1.4	2.0 ± 2.0	0.25 ± 0.46
	Chest tube placement	3.1 ± 1.4	3.3	3.0 ± 1.2	0.25 ± 0.71

Speedier operation, minimal blood loss, less requirement of stay in ICU or chest tube placement, faster narcotic-free recovery and earlier ambulation in one day pointed out the multifaceted surgical advantages of UniVATS. Because of more accessible to the lung through subcutaneous fat with less burden in high-risk patients^23^, the relative indications for UniVATS in thoracic surgery include high BMI or compromised cardiopulmonary function^24^. Our case of TOF exemplifies the safety and feasibility of this technique. However, the hospital stay might be longer (9.8 ± 2.3 days) because of Taiwan National Health Insurance allows patients to hospitalize longer for recovery. The intercostal nerve blockade and drainless protocol further expedited the recovery. Hung *et al*.^25^ showed that the popularity of regional blockades reduced postoperative narcotics requirement, which impeded lung expansion and obstructed recovery. Less access trauma of UniVATS together with intercostal nerve blockade make narcotic-free recovery possible in our institute. On the other hand, the placement of the chest tube potentially caused wound pain and precluded patients from early ambulation and discharge. Yang *et al*.^26^ reported that drainless UniVATS was safe with reduced postoperative pain and hospital stay. Therefore, this protocol was routinely practiced in our institute. The halo-gravity traction and halo-femoral traction commonly used in the staged operations were not practiced in our cases, resulting in better comforts, faster recovery and fewer complications. For example, pressure sores, stiffness of the hip and knees due to the longer bed rest and more extended hospitalization had been reported^27^. Despite extremely severe preoperative curvatures, our study provided non-inferior surgical outcomes. UniVATS contributed to adequate correction with fewer complications and faster recovery. To the best of our knowledge, the benefits of UniVATS for anterior release outweighed the potential risks.

In addition to the minimal incision and faster recovery with comparable coronal and sagittal correction, UniVATS exceeds conventional multi-portal anterior approaches in view of techniques of demands. The single-port design of UniVATS has a simultaneous introduction of the instruments in line of the scope, which offers another advantage to surgeons with the parallel visual field as an open approach. The conventional multi-portal VATS had a dihedral or torsion angle between the instruments and the geometric plane of the scope, which represented an impediment to the depth visualization on the flat two-dimensional vision of monitors. On the contrary, in the case of UniVATS, all the instruments are scattered on the geometric plane of the scope that preserves the depth of intraoperative visualization. To maximize the advantages, it is suggested^28^ that keeping the geometric plane of the scope as close as possible to the plane of the instruments, producing a surgical field of view that equal to the open thoracotomy when facing the patient’s anterior chest. We avoided setting incision too anterior because the mediastinum, deflated lung, and surrounding vessels limited the accessibility to the spine^29^. As a result, the spatial advantage of UniVATS flattens the substantial learning curve in conventional multi-portal VATS that requires enormous time and effort to overcome. The previous study^24^ showed that UniVATS was not so different from an open approach, and the transition to UniVATS could be less traumatic than to a conventional multi-portal VATS.

Although the safety and therapeutic effects of UniVATS on anterior release in severe scoliosis patients were shown in our study, some limitations should be considered, including the small sample size, relatively short follow-up and lack of postoperative scoliosis-specific outcomes questionnaire (SRS-22) to quantify the patient’s subjective satisfaction. Because of high population coverage of the National Health Insurance, scoliotic patients could be detected and treated earlier in Taiwan. The cases of severe scoliosis curves were so rare that our sample size was relatively small. Moreover, the anterior releasing procedures to the level far from the incision would be incomplete due to larger access angle. Therefore, the maximum release-level was limited to four or five levels according to the size of chest cavity. The development of angled surgical instruments potentially facilitates the accessibility to distal discs. The long-term follow-up and prospective clinical trials at larger scale should be conducted. Despite these limitations in the study design, our study did offer convincing evidence and valuable information for this novel approach in the management of severe scoliosis.

In summary, this is the first study to apply the novel UniVATS in the field of orthopaedics to treat patients with severely stiff thoracic scoliosis. The safety and efficacy of UniVATS have been proven even in those with higher cardiopulmonary risks. Compared with conventional approaches for anterior release, UniVATS contributes to comparable surgical outcomes with minor access trauma, speedier operation with minimal blood loss, less requirement of stay in ICU or chest tube placement, faster narcotic-free recovery and earlier ambulation within one day. UniVATS could be an optimal way to treat severe thoracic scoliosis in light of the advantages of less pain, faster recovery and superior cosmetic results without significant complications.
